# From jhum to broom: Agricultural land-use change and food security implications on the Meghalaya Plateau, India

**DOI:** 10.1007/s13280-015-0691-3

**Published:** 2015-08-09

**Authors:** Rabi Narayan Behera, Debendra Kumar Nayak, Peter Andersen, Inger Elisabeth Måren

**Affiliations:** Department of Geography, North-Eastern Hill University, Shillong, 793022 India; Department of Geography, University of Bergen, Fosswinckelsgate 6, 5007 Bergen, Norway

**Keywords:** Cash-cropping, Farming system, Land-use change, Northeast India, Population pressure, Slash and burn

## Abstract

Human population growth in the developing world drives land-use changes, impacting food security. In India, the dramatic change in demographic dynamics over the past century has reduced traditional agricultural land-use through increasing commercialization. Here, we analyze the magnitude and implications for the farming system by the introduction of cash-cropping, replacing the traditional slash and burn rotations (jhum), of the tribal people on the Meghalaya Plateau, northeast India, by means of agricultural census data and field surveys conducted in seven villages. Land-use change has brought major alterations in hill agricultural practices, enhanced cash-cropping, promoted mono-cropping, changed food consumption patterns, underpinned the emergence of a new food system, and exposed farmers and consumers to the precariousness of the market, all of which have both long- and short-term food security implications. We found dietary diversity to be higher under jhum compared to any of the cash-crop systems, and higher under traditional cash-cropping than under modern cash-cropping.

## Introduction

Many South Asian countries have witnessed a shift from traditional to high-value crops with associated change in land-use patterns over the past decades (Tipraqsa and Schreinemachers 2009), including the Indian Himalayan states (Saxena et al. 2005; Rahut et al. 2010). In developing countries, several studies have recognized the direct link between agriculture and food security of farm households, as well as the impacts of commercialization of agriculture on local food security. However, there is no unanimity in regards to the nature of this impact on food security at the household level. While a few studies (von Braun and Kennedy 1986, 1987; Kennedy 1994; Tipraqsa and Schreinemachers 2009) reveal generally positive impacts, others (Patnaik 1996; Dauvergne and Neville 2010; Patel-Campillo 2010; Anderman et al. 2014; Temudo and Manuel 2014) found negative impacts, either directly or indirectly, or on a short- or long-term basis. These studies show large variations in food security implications caused by commercialization of agriculture depending on context and time. The difference between the two stated positions is mainly reflecting a difference in approach, indicators used, and, more importantly, the diversity of contexts. Each cash-crop system has its unique effects depending on the factors such as labor requirements, economies of scale, capital investment, and gestation period (von Braun and Kennedy 1986, 1987). Those who are in support of commercial agriculture for household food security have mostly ignored the issues of sustainability and the loss of agro-biodiversity. Only few (but see Choudhury 2005; Ducourtieux et al. 2006) have examined the transition process directly from shifting cultivation to market-oriented cash-cropping, which is the prime concern of the present study. Further, food security under commercialization has scantly been researched in the northeastern states of India (Hussain 2004; Basu et al. 2006; MSSRF-WFP 2008; Menon et al. 2009). The agricultural landscape in rural Meghalaya, a hill state in northeastern India, has over large areas changed from shifting cultivation directly to commercial cropping, possibly impacting the local agriculture, food system, and the hill environment, with potential implications for food security as the food habits are closely linked to the agricultural system. As this process is likely to be further intensified in the coming years due to population growth, reduced fallow cycles, and the government’s encouragement of commercial crops, we find it timely to analyze its possible implications for food security of the region.

The Meghalaya Plateau is unique in its bio-geophysical and socio-cultural aspects: Firstly, its location is remote and only recently connected with the national infrastructure grid. Secondly, the direct transition from jhum cultivation to cash-cropping is relatively rarely documented in the published literature and is different from the Boserupian, gradual intensification of cultivation cycles. The jhum cultivation system differs in terms of both land-use and livestock composition, as the farmers do not have cows and buffalos, unlike in large parts of India, but pigs as their main livestock, leading to fundamentally different farming systems. Culturally, the region represents different food preferences compared to the rest of India, primarily by traditionally having less preference for dairy products and common pulses. Finally, land-use is greatly influenced by the hilly terrain, imposing agro-ecological constraints on the region; Meghalaya has not seen the introduction of commercial cereals and other cash-crops until now, in contrast to the farmers of the central Ganges plain who experienced this transition during the green revolution. Here, we aim to (1) analyze agricultural land-use change on the Meghalaya Plateau and (2) explore whether, and to what extent, land-use change has altered the food system, and ultimately the food security, for the people of Meghalaya.

## Materials and methods

### Study site

**Fig. 1:**
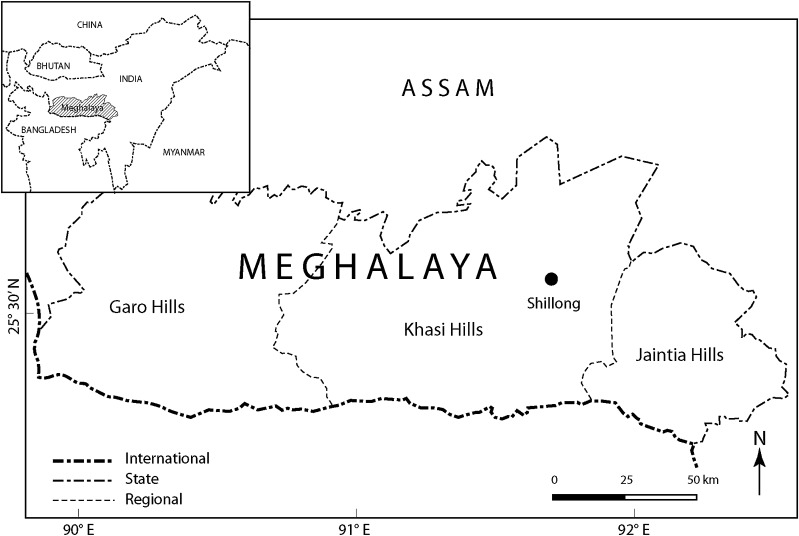
Map showing the three regions—Garo Hills, Khasi Hills, and Jaintia Hills—of the state of Meghalaya, with *inset map* showing the location of Meghalaya in the Northeast of India

The Meghalaya Plateau, a highly dissected plateau between the Brahmaputra Valley to the north and the Bangladesh plain to the south, is located in the state of Meghalaya in the northeastern part of India, between 25°00′N to 26°10′N and 89°45′E to 92°47′E (Fig. 1). The altitude ranges from 50 to 1950 m asl, with the highest peak, the Shillong Peak, situated centrally in the plateau of the Khasi Hills. The plateau enjoys monsoon climate, and the climate varies significantly with varying altitude and physiography (Department of Agriculture 2006). The rainy season (monsoon) lasts from mid-June to mid-September. The average annual rainfall of the plateau is around 1200 mm (however, the two wettest places on earth, Mawsynram and Cherrapunji, are located in the southern part of the plateau) (Gopalakrishnan 1995; Soja 2004). July is the hottest month with a mean temperature of 28°C, and January the coldest with a mean of 5°C, recorded in the state capital Shillong.

The annual population growth rate was 2.78% (2001–2011), and the population in 2011 was 2 964 007, with the tribal population constituting 86% of the total population (Census of India 2011). A number of tribes—chiefly the Khasi, Garo, and Jaintia—all of whom practice a matrilineal social system, traditional practices, and institutions live in this area. A major proportion (79%) of the total population lives in small villages of rural Meghalaya. Close to 60% of the total urban population is confined to Shillong (Census of India 2011). People’s livelihoods revolve around agriculture and other primary activities. They cultivate a variety of crops classified into two broad groups: subsistence and cash-crops. However, this classification is not completely clear since some subsistence crops also have a commercial role. The first group includes millet, rice, maize, soya, tubers, oilseeds, spices, vegetables, and leafy vegetables for household consumption. Crops like broom grass (*Thysanolaena maxima*, hereafter called ‘broom’),^1^ areca nut, rubber, cashew nut, black pepper, tea, coffee, and various fruits are grown for commercial purposes, finding their way into local, national, and international markets. The plateau suffers from poor infrastructure facilities, particularly of roads and communications, and nearly half of all the villages are not connected by all-weather roads. The plateau has 11 administrative districts that fall into three distinct regions—Garo Hills, Khasi Hills, and Jaintia Hills—on the basis of their regional socio-cultural and bio-physical characteristics (Fig. 1).

jhum denotes a form of shifting cultivation practice common in northeast India which does not include the shifting of settlement; rather, it is limited to the shift of cropping plots only. The jhum system was up until recent times the source of food, livelihood, the dominant land-use practice, and the way of life for all tribal groups in the hills of northeast India (Ramakrishnan 1993). The system includes a variety of foods, including cereals (millet, maize, and rice), vegetables, legumes, tubers, oilseed, and leafy vegetables, and non-food items like fiber for clothes and thatch grass for house construction. This age-old practice has undergone substantial changes and modifications with regard to management practices and extent of area, and land under jhum has been declining over the last five decades (Census of India 1983; Ministry of Agriculture, Government of India 1983). According to Tiwari (2003), with increasing population density, the proportion of fallow land is falling rapidly, and, as a consequence, the length of the fallow cycle, so essential for the regeneration of soil fertility, has been drastically reduced from 13–15 years some 25 years ago to a mere 3–4 years at the present day.

### Data collection

We used agricultural land-use data collected by the Directorate of Agriculture, the Directorate of Economics, Statistics and Evaluation, and the Directorate of Soil and Water Conservation, Government of Meghalaya in Shillong. Acreages under five subsistence crops (rice, millet, maize, soybeans, and sweet potato), eight traditional cash-crops (areca nut, citrus, turmeric, ginger, banana, pineapple, tapioca, and black pepper), and five modern cash-crops (potato, rubber, cashew nut, tea, and coffee) were selected. Cash-crops are classified into two categories; *traditional* and *modern*. Traditional cash-crops are those that are or have been known to be cultivated by the local people as subsistence crops, though many of these are now commercialized. Modern cash-crops have never been part of the traditional cultivation of this region and therefore only gradually appear in the statistical records. The year 1973–1974 was chosen as the base year for understanding changes in land-use and crops, based on the availability of data. However, the base year of 1973–1974 could not be used for all cash-crops, e.g., ginger, which was not recorded until 1985–1986. Multiple base years have therefore been used for modern cash-crops, depending upon their appearance in the records: rubber: 1957–1958 to 2010–2011; black pepper: 1959–1960 to 2010–2011; cashew nut and coffee: 1962–1963 to 2010–2011; areca nut, turmeric, banana, and potato: 1973–1974 to 2010–2011; citrus and pineapple: 1974–1975 to 2010–2011; ginger: 1985–1986 to 2010–2011; and tea/strawberry: 1997–1998 to 2010–2011. This set of macro land-use data was used to analyze changes in cropping patterns at regional level over the recent decades.

**Fig. 2:**
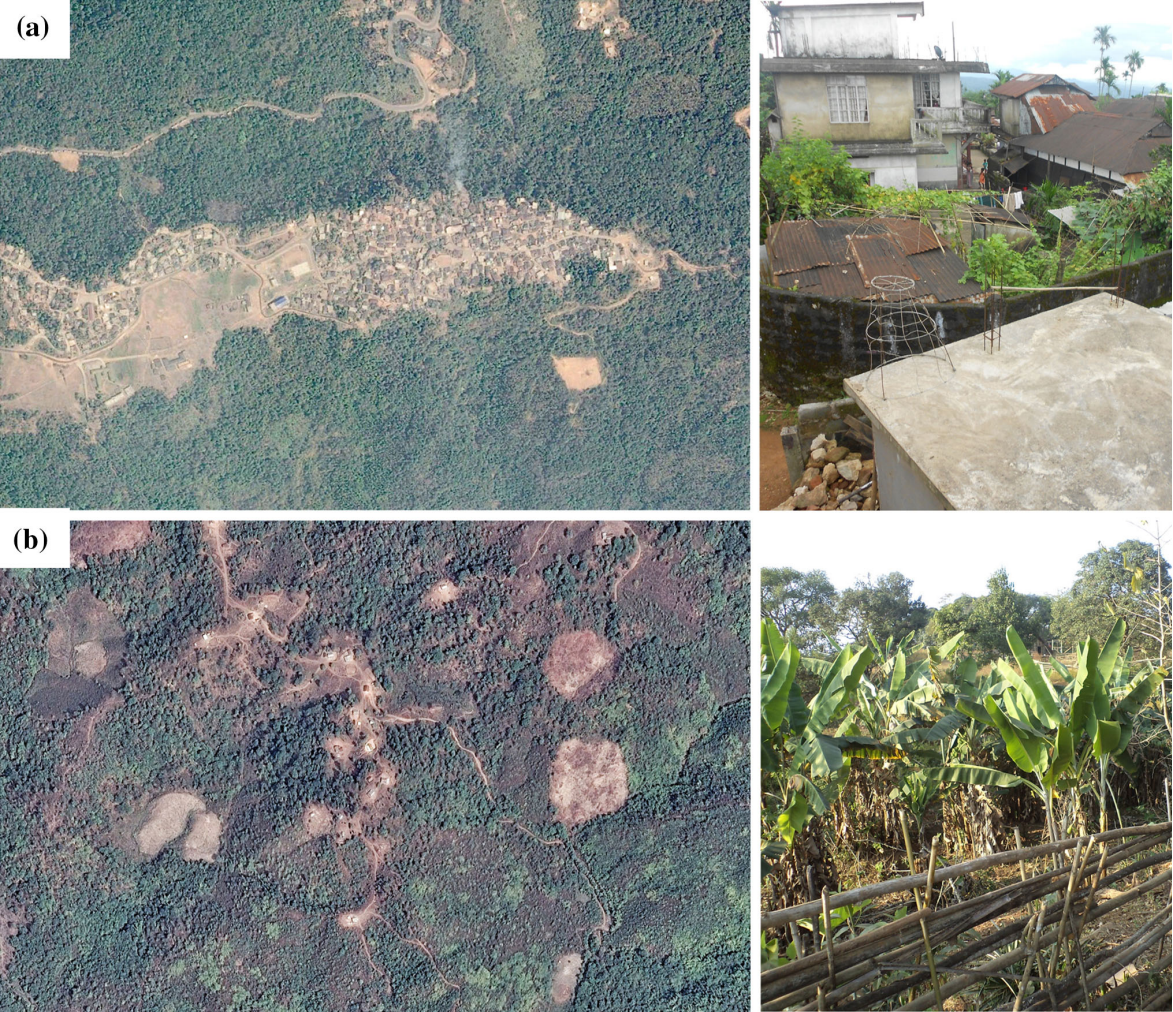
Spacing between housing units in the state of Meghalaya, India: (**a**) little space between housing units for kitchen gardens in areca nut plantation areas (Nongtalang village) and (**b**) ample space between housing units generally used as kitchen garden in jhum areas (Mawrynniaw village). *Sources* Google Earth viewed from 1300 m distance, photo (**a**) taken on 9/13/2011 and photo (**b**) taken on 10/2/2010, photos to the right taken by the first author in 2013

Two broad farming systems^2^ have been identified in the plateau; the jhum-based and the cash-crop-based farming systems. The cash-crop-based farming system can further be divided into traditional and modern. On the basis of the above classification, we selected seven villages for our field study and field work was carried out in October and November of 2013. Mawrynniaw (Fig. 2b) (25°28′19″N and 91°04′41″E) (coded jhum I) and Jongchetpara Songma villages (25°30′26″N and 90°02′18″E) (coded jhum II) represent the jhum farming system, where a variety of food crops were cultivated chiefly for domestic consumption. These two villages were remotely located, and the total populations were 192 (32 households) and 306 (59 households), respectively. The next three villages—Kshaid (25°12′34″N and 91°46′05″E), Nongtalang (Fig. 2a) (25°12′32″N and 92°04′06″E), and Thadnongiaw (25°44′19″N and 92°03′51″E)—represent traditional cash-crop-based farming systems, producing broom, areca nut, and ginger. Nongtalang and Thadnongiaw villages were connected with a concrete road, and the total populations of these villages were 2401 (391 households) and 622 (102 households), respectively, while Kshaid was not connected with a concrete road, with a total population of 287 (59 households). The last two villages—Machokgre (26°03′22″N and 91°50′25″E) and Sohliya Mawthoh (25°44′58″N and 91°59′33″E)—represent modern cash-crop-based farming systems, rubber and tea/strawberry plantations, respectively, and are well connected with road access and with populations of 127 (21 households) and 259 (45 households), respectively. We used participant observation, Participatory Rural Appraisal (PRA), and focus group discussions to gain insights into changing food habits, agro-biodiversity, and sources of food in these seven villages. The first author revisited these seven villages several times during 2010–2014. Additionally, he had 1 year of prior involvement with participatory research on the implications of cash-crop plantations and best practices of shifting cultivation in the region in association with the Regional Centre, National Afforestation and Eco-Development Board, Ministry of Environment and Forests, Government of India. A dietary module was prepared, based on the understanding through participant observation, in-depth interviews, focus group discussions, and informal conversations with villagers, to represent the frequency of food consumption at the farming system level. PRAs were conducted with the household heads in respective villages with the help of the dietary module.

The official land-use data are based on estimations and methods can vary between institutions, hence giving rise to errors. The estimations were carried out by trained personnel, using parameters like quantity of seeds used, land cleared for cultivation, and estimation of area or plots to be grown for certain crops at village levels for the entire state. Small plots, intercropping, and limited accessibility add to the difficulty of data collection. However, we argue that the official data can be useful to indicate land-use trends over time and the land-use data are also important in a situation where there are no other sources of data available as the region is categorized as ‘an Indian state with no land records and cadastral survey.’ The food consumption data collected at village level through PRA present an aggregate picture at village level which ignores intra-household differences. This information gathered using qualitative methods is simple and feasible at community level, but not generalizable beyond the community level (Chung et al. 1997). However, we observed similarity in intra-household food consumptions pattern within the studied villages, irrespective of differences at household level during pre-PRA observations. The community-level homogeneity of food consumption pattern is an outcome of a broadly similar resource base and shared cultural codes in the region.

## Results

### Demography and agricultural land-use change

**Fig. 3:**
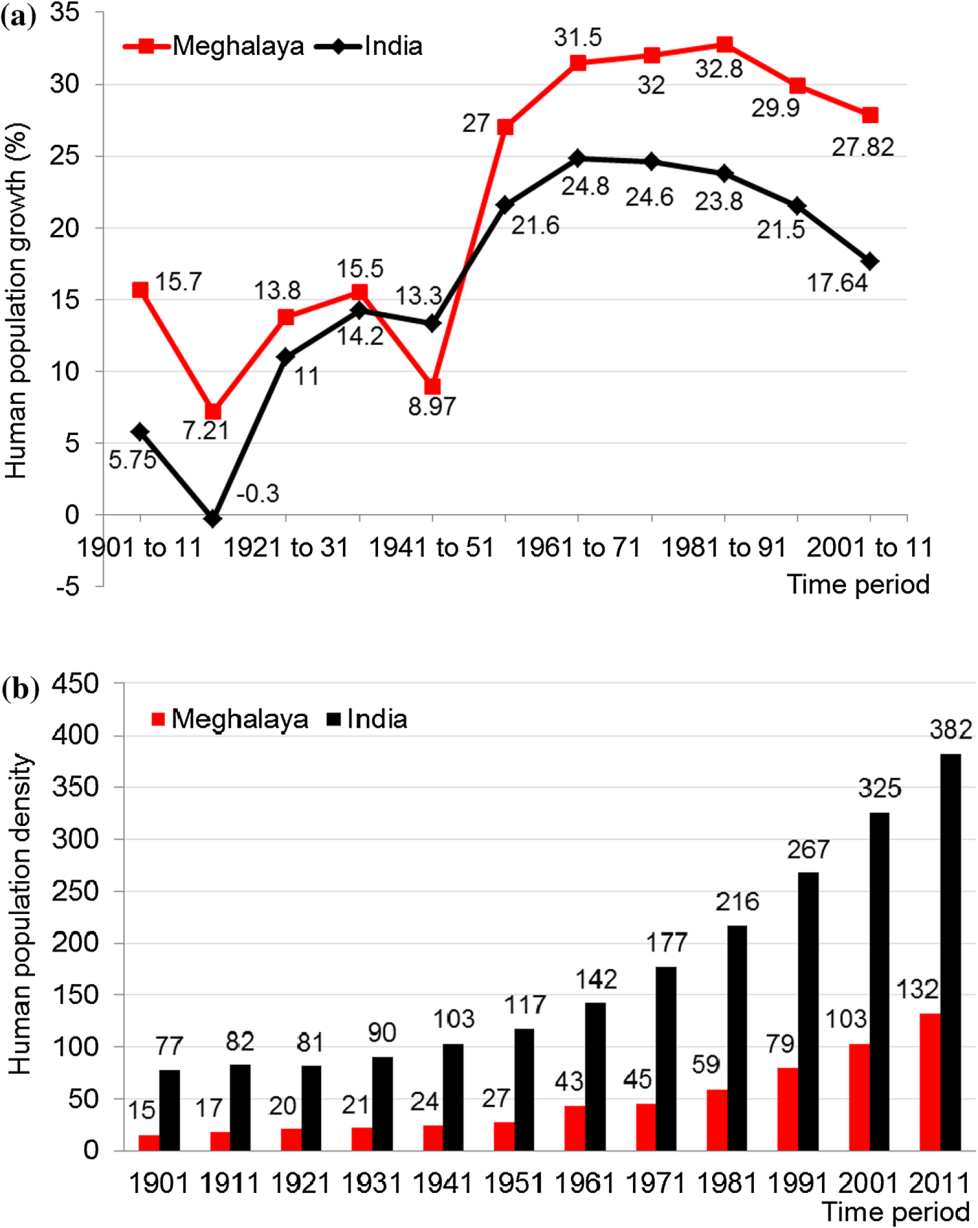
Human population (**a**) growth (percent) and (**b**) density (persons per square km) in the state of Meghalaya (*red*) and for the whole of India (*black*), over 11 decades from 1901 to 2011. *Source* Census of India

There has been a significant rise in population and population density in the state of Meghalaya since 1901 (Fig. 3). The population density increased from 15 persons km^−2^ in 1901 to 132 in 2011. Moreover, the population growth in Meghalaya was much higher than the national average in the twentieth century, and within Meghalaya it was the highest in the Jaintia Hills.

**Fig. 4:**
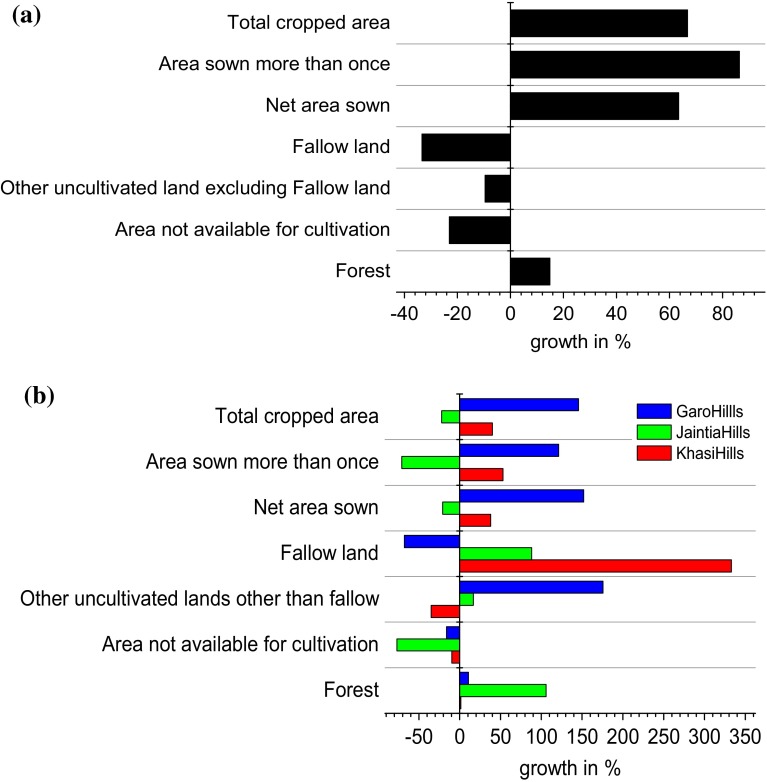
Land-use/cover change in the state of Meghalaya, India: (**a**) overall change of major agricultural land-use categories and (**b**) regional change of major agricultural land-use categories, over 37 years (from 1973–1974 to 2010–2011). *Source* As for Table 1

The substantial increase in area under cultivation has taken place at the cost of area under non-agricultural use, including fallow lands, indicating a reduction in the jhum cycle and/or a switch to other agricultural practices (Fig. 4a). Interestingly, the reduction of fallow land area is largely confined to regions where jhum is practiced extensively, such as in the Garo Hills. In the Khasi and Jaintia Hills, where the proportion of jhum land is far less, there has been a sharp increase in fallow land (Fig. 4b). The extent of forested land has also increased across regions and is most pronounced in the Jaintia Hills, though this growth is partly attributed to an increase in area under perennial cash-crops such as bamboo plantations (unfortunately categorized under “forested land”). There has been an overall decrease in area under subsistence crops (except that of soybean) across regions, with the exception of the Garo Hills where jhum is still the predominant land-use practice, and a substantial increase in area under cash-crops, irrespective of regional differences (Table 1). Areas under both food and cash-crops show an increase in the Garo Hills. Diffusion of many modern cash-crops (e.g., rubber and cashew nut) is also high in the Garo Hills. In contrast, area under traditional crops (except citrus) witnessed substantial increase, but the rise in modern cash-crops in the Khasi Hills is less pronounced compared to the Garo Hills. There has been little penetration of modern cash-crops into the Jaintia Hills.

**Table 1:** Change in percent and in hectares (parenthesis) in area under subsistence crops and cash-crops, in the three regions of the state of Meghalaya, India. Time periods: a = 1957/1958–2010/2011, b = 1962/1963–2010/2011, c = 1969/1970–2010/2011, d = 1973/1974–2010/2011, e = 1974/1975–2010/2011, f = 1984/1985–2010/2011, and g = 1997/1998–2010/2011

Crop type	Time period	Khasi Hills	Jaintia Hills	Garo Hills
Rice^a^	d	−39.7 (−15 309)	−34.0 (−6385)	31.2 (17 315)
Maize^a^	d	−18.8 (−1802)	−92.7 (39 216)	17.5 (960)
Millet^a^	d	−68.3 (−926)	−63.1 (−278)	92.9 (790)
Sweet potato^a^	d	−23.8 (−664)	0.5 (6)	26.1 (192)
Soybean^a^	d	1240.0 (372)	103.9 (209)	280.0 (196)
Total food grains^a^	d	−35.7 (−17 682)	−33.3 (−7832)	34.8 (21 776)
Areca nut^b^	d	25.6 (1202)	67.9 (690)	2028.1 (6693)
Citrus^b^	e	41.8 (1703)	42.3 (320)	80.0 (1352)
Turmeric^b^	d	364.1 (193)	32.7 (276)	12.8 (64)
Ginger^b^	f	52.5 (614)	245.7 (231)	27.6 (1589)
Banana^b^	d	101.1 (1224)	−24.9 (−116)	220.9 (2762)
Pineapple^b^	e	29.9 (1213)	−84.3 (−398)	153.6 (3192)
Tapioca^b^	d	8.8 (91)	(NA)	243.2 (2165)
Black pepper^b^	c	102650.0 (410.6)	(NA)	(NA)
Potato^c^	d	10.3 (1570)	−82.4 (−880)	12.0 (78)
Rubber^c^	a	23119.7 (1831.0)	−(611)	15688.7 (2347)
Cashew nut^c^	b	−100.0 (−20)	(NA)	13415.6 (8586)
Tea^c^	g	−(1188)	(NA)	227.5 (421)
Coffee^c^	b	469.7 (77.5)	(NA)	23650.0 (94.6)

Computed from *Statistical Abstract of Meghalaya*, 1978, GoM, Directorate of Economics, Statistics and Evaluation, Shillong, p. 84 and *Reports on Area, Production & Yield of Agricultural Crops, 2010*–*2011*, GoM, Directorate of Agriculture, Shillong, Meghalaya. Base years and current year compiled from *Statistical Abstract of Meghalaya*, 1978, GoM, Directorate of Economics, Statistics and Evaluation, Shillong, p. 84 and *Reports on Area, Production & Yield of Agricultural Crops, 2010*–*2011*, GoM, Directorate of Agriculture, Meghalaya, Shillong

Base year figures for rubber, cashew nut, coffee, and black pepper are provided by Directorate of Soil and Water Conservation, Shillong. Crops like broom and bay leaf are still considered forest products in the state and area under these crops are not available at the government department

^a^Subsistence crops

^b^Traditional cash-crops

^c^Modern cash-crops

**Fig. 5:**
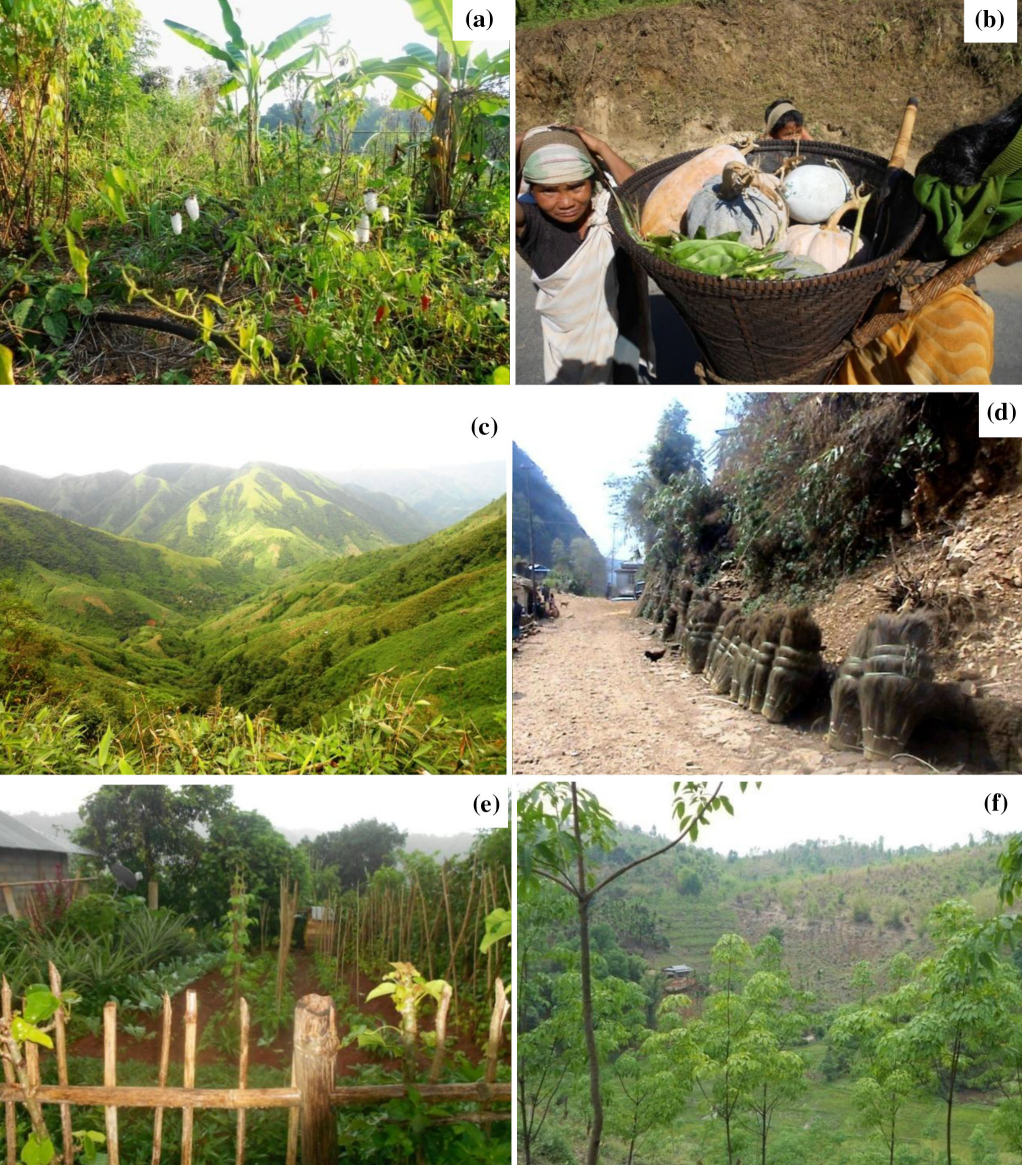
Pictures form the study area in the state of Meghalaya in India, showing (**a**) a diversity of food crops cultivated under the jhum system, (**b**) women returning from jhum fields with traditional food baskets in Mawryniaw village, (**c**) broom cultivation at large scale, close to Kshaid village (**d**) broom for sale by the road side, Kshaid village, (**e**) space between housing units used as kitchen garden in Thadnongiaw village, and (**f**) recently introduced rubber plantation, Machokgree village (all photos by the first author)

Villages still practicing jhum cultivate more varieties of food crops (Table 2), and villages switching over to cash-crops are abandoning cultivation of food crops altogether or with marginal production of one kind of cereal, as seen in Nongtalang village, which is dependent on areca nut plantations (Table 2a). In Machokgre village where they produce rubber extensively, we saw an extreme situation where even vegetables, tubers, and leafy vegetables were no longer cultivated or collected from the forest (pers. obs.). Broom, areca nut, rubber, and ginger were grown as mono-crops in the respective villages (Fig. 5).

**Table 2:** (a) Agro-biodiversity (number of species used) under different crop systems and (b) food sources in different farming systems in the state of Meghalaya, India, based on the 2013 field surveys in 7 villages (2 under jhum, 3 under traditional cash-cropping, and 2 under modern cash-cropping). Mode of food acquisition: a = own production, b = hunting/fishing in adjacent forests/rivers, c = gathering, d = public distribution system, and e = market

Farming system	Cereals	Tubers	Vegetables	Leafy vegetables	Spices/oilseed	Pulses
(a)						
jhum: I	3	6	11	6	6	1
jhum: II	3	6	15	4	4	1
Traditional: Broom	0	1	0	2	0	0
Traditional: Areca nut	0	1	0	0	0	0
Traditional: Ginger	2	2	8	11	2	0
Modern: Rubber	0	0	0	0	0	0
Modern: Tea/strawberry	1	2	8	0	2	0

Farming system	Cereals	Tubers	Vegetables	Leafy vegetables	Spices/oil seed	Pulses	Wild fruit/vegetables	Meat	Fish
(b)									
jhum I	a	a	a	a, c	a	a	c	b, e	b, e
jhum II	a	a	a	a, c	a	a	c	b, e	b, e
Traditional: Broom	d, e	e	e	c	e	e	e	b, e	e
Traditional: Areca nut	d, e	e	e	c	e	e	c, e	e	e
Traditional: Ginger	a, d, e	a, e	a, e	a, e	a, e	e	e	b, e	b, e
Modern: Rubber	e	e	e	e	e	e	e	e	e
Modern: Tea/strawberry	a, d, e	a, e	a, e	a, e	e	e	e	e	b, e

### Changing food consumption

Rice has replaced the consumption of millet and maize as the main staple in villages switching over to cash-crop cultivation (Table 3). Further, people living under cash-crop regimes mostly depend on white rice, unlike people of the jhum systems who consume three cereals, millet, maize, and brown rice. People in cash-crop villages occasionally consume wheat flour which is not produced locally, and the consumption of potato is rapidly replacing traditional tubers such as taro, sweet potato, tapioca, and yam. People mostly depend on nearby forests for traditional fruits, both in jhum and cash-crop villages. However, the availability of wild fruits is higher in the jhum areas than in the cash-crop areas, as the forests are replaced by cash-crops, thereby reducing the availability of wild fruits and vegetables. The protein intake has not improved, in terms of quantities of pulses and animal source foods consumed, in the villages which switched to cash-cropping. Villagers of jhum mostly depend on their own livestock and wild meat hunted from the forest and rarely purchase meat products from the market, while cash-cropping villagers depend on the market for animal proteins. There is no notable difference in the consumption of egg among the different food systems (Tables 2, 3); however, the difference is in terms of production mode of these protein sources; traditionally, farmers rear chicken and pigs at their homesteads for household consumption, while the cash-cropping farmers depend on industrial farm-raised chicken and pork. Local fish is consumed more in ginger areas, probably reflecting availability. The consumption of vegetables was lower in modern cash-crop areas than in jhum and traditional cash-crop areas. The consumption of leafy vegetables was common across all the food systems, but lowest in the rubber food system. Culturally, pulses (except ricebean *Vigna umbellata*) and milk products are not part of the indigenous food habit. However, ricebean is grown traditionally under the jhum system. In both traditional and modern cash-crop regimes, the dependence on market has increased. However, under traditional cash-crops, dependence on government subsidized food made available through public distribution system is considered an alternative to the shortage of cereals (Table 3).

**Table 3:** Food consumption patterns in different farming systems in the state of Meghalaya, India, where 0 = never, 1 = rarely, 2 = occasionally, 3 = sometimes, 4 = regularly, and 5 = daily; data based on field surveys in 7 villages (2 under jhum, 3 under traditional cash-cropping, and 2 under modern cash-cropping)

Item	Farming system						
	jhum I	jhum II	Broom	Areca nut	Ginger	Rubber	Tea/strawberry
Cereals							
Millet	4	3	0	0	0	0	0
Maize	4	4	1	1	1	1	1
Rice	4	4	5	5	5	5	5
Wheat	0	0	2	2	1	2	2
Tubers							
Taro	5	5	3	3	3	1	3
Sweet potato	4	4	1	2	1	2	1
Tapioca	4	4	1	1	1	1	3
Yam	4	4	1	1	2	2	2
Potato	1	2	4	5	5	5	5
Vegetable							
Leafy	5	4	5	5	4	3	3
Seasonal	4	4	4	3	4	3	4
Pulses and oilseed							
Red lentil	1	1	2	2	2	3	2
Horse gram	0	0	2	1	0	1	1
Rice bean	3	3	2	2	3	1	1
Soybean	3	2	1	1	1	1	1
Sesame	4	2	1	2	1	1	1
Animal products							
Wild meat	3	2	1	1	0	0	0
Pork	2	2	3	4	2	3	3
Beef	2	2	2	0	2	3	3
Chicken^L^	2	2	2	1	1	1	1
Chicken^F^	1	1	1	3	1	3	2
Egg^L^	3	3	3	1	3	1	1
Egg^F^	1	1	3	3	2	3	3
Fish^L^	3	3	1	1	4	2	2
Fish^F^	1	2	2	3	1	3	2
Milk	0	0	0	0	0	0	0

*L* local variety/wild and *F* farm-raised variety

## Discussion

### Evolution in crop regimes

The initial shift from jhum to cash-crops has been adopted as a coping strategy in the face of growing food insecurity. For example, the southern precipitous region (also known as a vernacular cultural region, *Ri*-*War*; Naken, 1961) adopted areca nuts, betel leaves, some indigenous tropical fruits (*Myrica nagi*, *Prunus nepalensis*, *Eleagnus khasianum*, *Flemingia vestita*, and *Docynia indica*), and citrus plantations in the mountains along the Indo-Bangladesh border, including the Cherrapunji area. One of the best examples of such a shift is broom, which was never cultivated previously, but was growing wild in the forests (Patel 1992; as cited in Tiwari and Kumar 2008) and only recently has been commercialized (Fig. 5). Till today, it is classified as a forest product by the district councils and the state government (Tiwari and Kumar 2008; Kharwanlang 2010). However, broom has become an important commercial crop and is now grown all over the state, though mostly confined to the warmer slopes of the central plateau. Commercial plantations of broom are commonly planted in abandoned jhum land with scanty soil (minimum soil cover where the regolith is partly exposed) across the plateau (Tiwari and Kumar 2008) and require the planting of rhizomes, cleaning, and weeding. Other traditional crops which have been chosen as cash-crops in the different parts of the plateau include ginger, banana, betel nut, betel vine, fruits, and a variety of spices, e.g., turmeric (*Curcuma longa*) has been adopted in some parts of the Jaintia Hills. These crops have been grown in the plateau for a long time but only recently been commercialized. Thus, the initial cash-crop system was far more location specific, characterized by local crops and a traditional knowledge base.

**Fig. 6:**
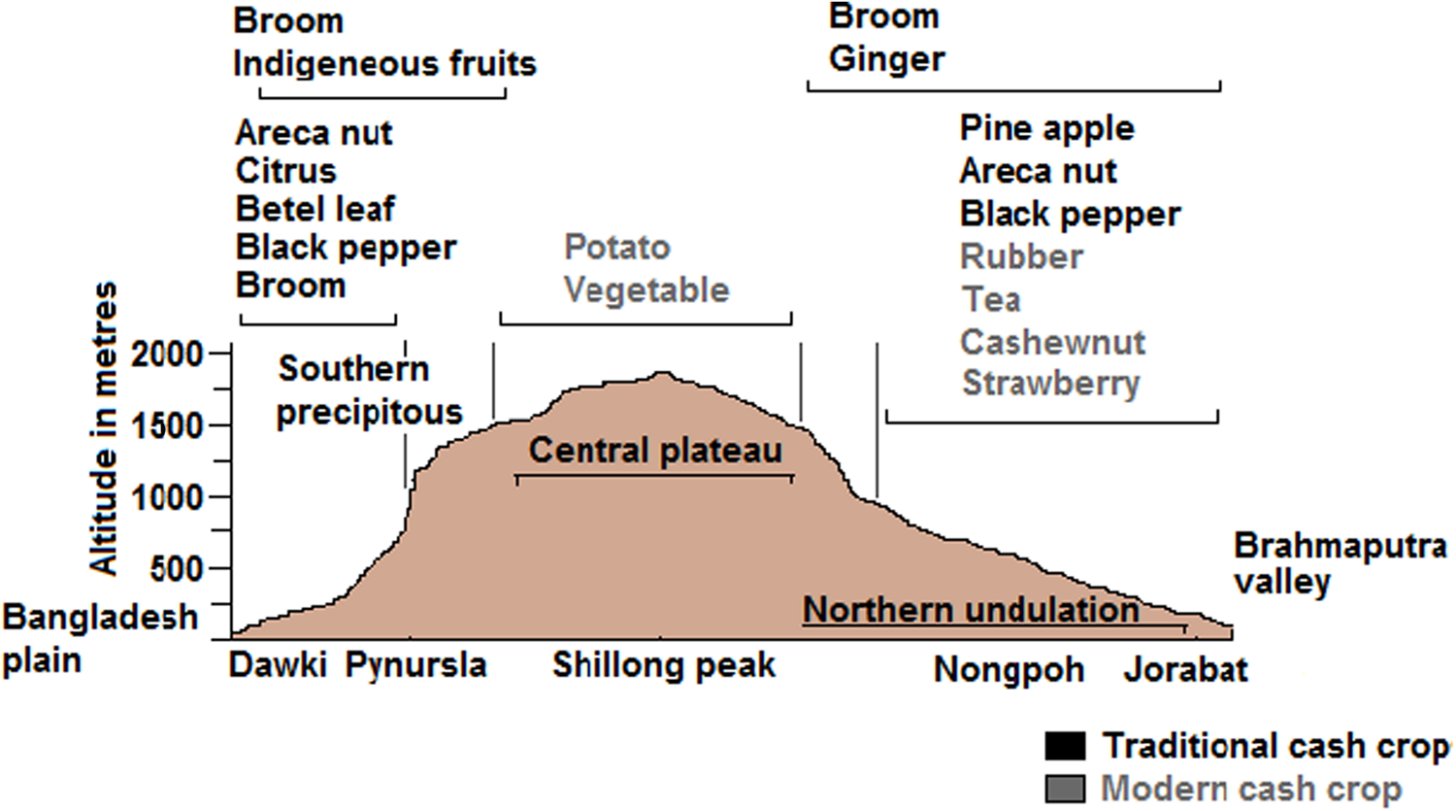
North–south cross section of the Meghalaya Plateau (Dawki–Pynursla–Shillong peak–Nongpoh–Jorabat) in India and the distribution of different cash-crops (traditional in *black font* and modern in *gray font*) over the major hypsographic regions: the northern undulation, the central plateau, and the southern precipitous (not to scale)

Potato was introduced in 1830 at higher elevations, particularly in the upper Shillong area (Nakane 1961). Later on, many modern commercial crops were introduced by various schemes and programs. Rubber, cashew nut, coffee, tea, and strawberry were introduced in 1957–1958, 1962–1963, 1962–1963, 1997–1988, and 2001, respectively. Like traditional cash-crops, the penetration of these modern cash-crops is also determined by altitude (Fig. 6) and regional socio-cultural settings. Rubber and cashew nut were planted at low altitude and in the foothills with relatively high temperatures in the Garo Hills and the northern undulating Khasi Hills. This shift took place in two evolutionary steps; first by commercialization of traditionally grown crops, followed by the introduction of modern cash-crops.

### Emergence of a new food system

Population growth has altered the man–land ratio and has made the traditional shifting cultivation practices increasingly non-viable. The Forest Survey of India Report (2011) states that the natural vegetation of the state has declined substantially, despite the reported annual increases in forest cover, and found the over-estimation of the forest cover largely due to the increase in bamboo cover. Scherr and Templeton (2000) attributed marginal growth in forest cover in hill areas with high population growth to an increase of managed forests like plantations. The food consumption data show that these areas provide less wild foods than natural forests and fallow land.

The land-use changes have caused new food systems to emerge in areas/villages which are shifting to cash-crops. Despite a considerable increase in the area under cultivation and yield of staple food grains aided by modern technology in the state, the present local food production has failed to meet the local demand of a growing population, and the dependence on food imports is on the rise. Regionally, the expansion of fallow land cover in the Jaintia and Khasi Hills is explained by the abandonment of jhum and a diversification to non-agricultural activities, while the situation is relatively better in the Garo Hills where the reduction in fallow land and growth of food crops parallels the growth in cash-cropping. This abandonment of agricultural activities is partly triggered by mining and quarrying activities. Moreover, local food systems are gradually becoming complex as food miles^3^ are rising over time. For example, grains are transported over long distances, from Punjab, Haryana, or Andhra Pradesh, located over 2000 km away, at a considerable cost. As the entire northeast of India is more prone to economic blockages, political strikes (*bandh*), and conflicts than the rest of the country, the food prices soar during such crises.

The denser housing structure under cash-crop regimes leads to the abandonment of traditional practices of keeping kitchen gardens and domestic livestock (pigs and chicken), characteristic of the subsistence food system. Commercialization of traditional fruits overtook the space of kitchen gardens in the broom food system. However, the ginger-based food system was different. Here, the space between houses was adequate and used widely as kitchen gardens.

### Consumption patterns and nutrition

We found that the production system has direct impacts on consumption patterns. Cash-crop production has reduced the diversity of staple foods consumed in cash-crop regimes, while diversity prevails (both in terms of production and consumption) in subsistence areas. Compared to rice, millet is rich in protein, amino acids, B-vitamins, minerals, and fiber. However, it contains very high amounts of phytate which severely restricts the bioavailability of iron and zinc (Padulosi et al. 2009). Wheat flour has been introduced recently, but the consumption is occasional as it is not used as a staple, unlike in northern India. Low intake of pulses is explained by inaccessibility to culturally preferred pulses, e.g., ricebeans, though red lentils and gram have made some inroads in cash-crop areas. *Tungrymbai* (fermented soybean) is traditional in the local food culture and a cheap source of protein (Sohliya et al. 2007), while green gram, horse gram, kidney beans, red lentils, and black gram, commonly used in the rest of India, have been introduced recently in local food markets but are less preferred and only occasionally consumed by local people. Ricebean (the preferred pulses) and *tungrymbai* are scarcely available in the local food market and rarely accessible because of high prices under cash-crop regimes. The loss of kitchen gardens and forests is also affecting the consumption of (leafy) vegetables and wild foods adversely in cash-crop areas. Particularly, the people in rubber plantation areas consume less leafy vegetables compared to those in ginger and areca nut plantation areas, where kitchen gardens and forests were present. Low consumption of animal products is attributed to the lack of livestock integration, particularly dairy farming, within the cash-crop system. Traditionally, the tribes of Meghalaya do not domesticate cattle, sheep, or goats, and thus the introduction of broom is of little consequence for livestock production. Such livestock could, however, enhance nutritional and economic benefits, as has been the case in the Ilam district of Nepal where broom plantations are successfully integrated with dairy farming (Takahatake 2001; Chapagain 2006). For Meghalaya, milk and pulses have great potential as nutritional substitution, as shown for other parts of India (NSSO 2012) and abroad.

### The ‘local’ versus the ‘imported’ (‘*Dkhar*’) dichotomy

Increased dependence on the market and food imports has created a recent food dichotomy; all food items available in local markets are classified into two types: ‘*dkhar*’ or ‘local.’ ‘*Dkhar*’ is a Khasi term widely used as a prefix for all imported foods, often denouncing that the item is relatively cheaper, less fresh, less tasty, and often the least preferred, compared to the ‘local’ produce. ‘*Dkhar*’ foods are accessible to the poor, while the ‘local’ is available to the privileged who prefer locally grown food, as seen elsewhere as well, irrespective of their food system (McEntee 2010). Despite this, as also Grossman (1993) shows in a study from Eastern Caribbean, people may prefer imported foods because of lower prices. This food dichotomy has recently evolved on the basis of location of production and is prevalent for many food items that used to be traditionally grown and consumed, but now are imported.

According to von Braun and Kennedy (1986, 1987), cash-crops have no adverse effects as long as the product/factor markets function perfectly and internal infrastructure is well developed. But neither market nor the compensation mechanisms function properly in the real world, particularly in developing countries (von Braun and Kennedy 1986, 1987). Mostly, the rural people of Meghalaya depend on the weekly market and nearby urban centers for essential commodities. The rural infrastructure is poor and this has significant adverse implications on food prices and accessibility as there are few proper roads, inadequate public transport, and, moreover, a vulnerability to landslides. For example, although the road distance between the two state capitals Guwahati (Assam) and Shillong (Meghalaya) is only 100 km, there is a significant difference, three to four times higher in Shillong, in the retail price of vegetables. This affects the availability and restricts the poor to access locally grown foods. Consequently, the most vulnerable groups are the poor in cash-crop regimes, particularly during times of political or natural crises.

The vagaries of the market also play its role in determining the price of cash-crops; one example is the drastic fall, 45–65%, in the selling price of areca nut over just 1 year (2011–2012). While this may be the case for just 1 year, the situation is becoming increasingly difficult for farmers who are highly vulnerable to price volatility, as there is no minimum support price for crops (except for soybean) produced by farmers of this region. There is also a lack of surveillance and monitoring by governmental institutions to ensure food safety and hygiene of food in the state, and as most of the food stuffs are now imported from outside the state, food safety is becoming a major area of concern, particularly for the market dependents.

### Ecological effects of land-use change

Transformation to commercial crops has enhanced mono-cropping, and the use of chemical fertilizers and pesticides, affecting the ecological sustainability and food security, both in the long and short run. Cultivation of commercial crops in the Meghalaya Plateau can have both positive and negative impacts on soil conservation and agricultural sustainability. Many of the cash-crops are tree crops or grown intercropped with trees, mimicking the soil conservation features of a natural forest, and balanced fertilizer inputs can substitute the fallow period. However, rubber growers reported that their plantations are more vulnerable to tropical cyclones and seasonal fires. Further, mono-cropping areas of areca nut and betel in the Khasi and Jaintia Hills are reported to be vulnerable to pests and diseases (Gassah 1998; Department of Agriculture, GoM 2006). Farmers in our study villages claimed that land used for broom becomes unsuitable for food crop production. One study also found that some areas of Meghalaya face adverse effects of broom cultivation on soil humidity as it dries up the land and consequently the streams and rivulets as well (Tribal Research Institute 2011).

## Conclusion

 Notwithstanding regional differences, commercial crops have made significant inroads on the Meghalaya Plateau of India, inducing a shift from subsistence farming to commercial, multi-cropping to mono-cropping, traditional to modern crops, and food to non-food crops. These changes have impacts on the production and consumption patterns, availability, sources, diversity of food, and the reliance on kitchen garden and markets, both in the short and long term. With subsistence shifting cultivation practice in the plateau becoming increasingly untenable in the wake of the increasing population pressure, a shift to cash-cropping has become inevitable. On the basis of the field survey conducted in the seven hill villages, we conclude that some, if not all, of these changes have negative implications on food security. This study reveals that a shift to commercial cropping does not necessarily translate into better nutrition in terms of greater consumption of vegetables, pulses, or animal source foods in cash-crop areas, compared to the traditional jhum areas. jhum and traditional cash-crop regimes promote greater intake of vegetables compared to modern cash-crop regimes, where a depletion of agro-biodiversity and kitchen gardens is reflected in the depletion of food quality at the household level. A diversity of vegetables is slowly going out of the food baskets in modern cash-crop systems, like in the rubber plantations where people completely depend on the market for vegetables. In spite of increased cash income in cash-crop regimes, the intake of pulses and animal products remains low and there is little difference in the consumption of animal products across the food systems. The nature of the traditional cash-crop system of the plateau is unique from an Indian, and even international, point of view (Bharadwaj 1985). It is largely based on commercialization of traditional crops, low capital investment, and use of traditional knowhow, highly dependent on community and family workforce, and is non-intensive, and the crop choices are guided by traditional land rights and customary laws. Contrary to traditional cash-crop systems, the modern cash-crop system is largely a governmental intervention aimed to stop the jhum system. It depends on government subsidies and requires modern skills and high capital investment. However, most of these prerequisites for agricultural commercialization are still lacking in this region. This is broadly due to the prevailing situation in India’s northeast including low socio-economic status of rural people, poor accessibility, lack of proper market links, low stability of cash-crop prices, limited value addition of crops, and small-scale production due to small and marginal land holdings. Our study shows that adopting cash-crops by tribes of northeastern India as a coping strategy to food insecurity and as an alternative to the traditional jhum system is insufficient to address the issues of dietary quality and availability under the prevailing agro-ecological, social, and market conditions. It is clear that agricultural commercialization alone has failed to bring much improvement in dietary diversity in rural Meghalaya. Indeed, a farmer in the less-developed world has to be food self-sufficient in staples to achieve household food security in a context where rural food markets are scarce, isolated, and poorly integrated with domestic food markets and suffers from poor infrastructure, on the one hand, and where farmers are confronted with price volatility and poor socio-economic development on the other hand. However, it is difficult to conclude categorically whether the net impacts of agricultural commercialization are purely negative or positive; hence, there is a need for further research to fill this pertinent knowledge gap.

## Footnotes

1 Broom is used for sweeping and cleaning purposes in India. In the title, the term *broom* is used as a metaphor to describe 'cash-crop,' while in rest of the paper it refers to broom grass plantations.

2 A ‘farming system’ is defined as a population of individual ‘farm systems’ (a ‘farm system’ refers to an individual farm with specific resource endowments, family circumstances, and existing social, economic, and institutional environment and is organized to produce food and to meet other household goals, through a range of activities, including interdependent gathering, production, post-harvest processes, livestock keeping, fishing, agro-forestry, hunting, gathering activities, and off-farm incomes) that have broadly similar resource bases, enterprise patterns, household livelihoods, and constraints and for which similar development strategies and interventions would be appropriate (Dixon et al. 2001, pp. 8–9).

3 ‘Food miles’ is defined as the distance an item of food travels between where it is produced and where it is finally consumed, with each added mile decreasing the freshness of the food and, in some cases, increasing its carbon footprint’ (Paarlberg 2010, p. 194).
